# 22q11.2 Deletion Syndrome: Impact of Genetics in the Treatment of Conotruncal Heart Defects

**DOI:** 10.3390/children9060772

**Published:** 2022-05-25

**Authors:** Carolina Putotto, Flaminia Pugnaloni, Marta Unolt, Stella Maiolo, Matteo Trezzi, Maria Cristina Digilio, Annapaola Cirillo, Giuseppe Limongelli, Bruno Marino, Giulio Calcagni, Paolo Versacci

**Affiliations:** 1 Pediatric Cardiology Unit, Department of Pediatrics, Obstetrics and Gynecology, “Sapienza” University of Rome, Policlinico Umberto I, 00161 Rome, Italy; flaminia.pugnaloni@gmail.com (F.P.); unolt.marta@gmail.com (M.U.); stella_maiolo@yahoo.it (S.M.); bruno.marino@uniroma1.it (B.M.); paolo.versacci@uniroma1.it (P.V.); 2 Department of Pediatric Cardiology and Cardiac Surgery, Bambino Gesù Children’s Hospital, IRCCS, 00165 Rome, Italy; matteo.trezzi@opbg.net (M.T.); giulio.calcagni@opbg.net (G.C.); 3 Genetics and Rare Diseases Research Division, Bambino Gesù Children’s Hospital, IRCCS, 00165 Rome, Italy; mcristina.digilio@opbg.net; 4 Inherited and Rare Cardiovascular Disease—Pediatric Cardiology Unit, Monaldi Hospital, AORN Colli, 80131 Naples, Italy; cirilloannapaola@gmail.com; 5 Inherited and Rare Cardiovascular Diseases, Department of Translational Medical Sciences, University of Campania “Luigi Vanvitelli”, Monaldi Hospital, 80131 Naples, Italy; limongelligiuseppe@libero.it

**Keywords:** 22q11.2 deletion syndrome, conotruncal heart defects, perioperative management, cardiac surgical outcome

## Abstract

Congenital heart diseases represent one of the hallmarks of 22q11.2 deletion syndrome. In particular, conotruncal heart defects are the most frequent cardiac malformations and are often associated with other specific additional cardiovascular anomalies. These findings, together with extracardiac manifestations, may affect perioperative management and influence clinical and surgical outcome. Over the past decades, advances in genetic and clinical diagnosis and surgical treatment have led to increased survival of these patients and to progressive improvements in postoperative outcome. Several studies have investigated long-term follow-up and results of cardiac surgery in this syndrome. The aim of our review is to examine the current literature data regarding cardiac outcome and surgical prognosis of patients with 22q11.2 deletion syndrome. We thoroughly evaluate the most frequent conotruncal heart defects associated with this syndrome, such as tetralogy of Fallot, pulmonary atresia with major aortopulmonary collateral arteries, aortic arch interruption, and truncus arteriosus, highlighting the impact of genetic aspects, comorbidities, and anatomical features on cardiac surgical treatment.

## 1. Introduction

In the last 30 years, the continuous progress in fetal diagnosis, cardiac surgery and perioperative management allowed a significant reduction in mortality and morbidity, even for the most complex congenital heart diseases (CHDs) [1,2]. Despite a substantial improvement in the cardiac surgical prognosis of patients with cardiac defects, nowadays, it is well established that the clinical and cardiac postoperative outcome of CHDs can be adversely affected by the coexistence of a genetic syndrome [3]. This may depend on both the specific associated cardiac phenotype and the concomitant extracardiac comorbidities related to the syndrome [4,5].

**Figure 1:**
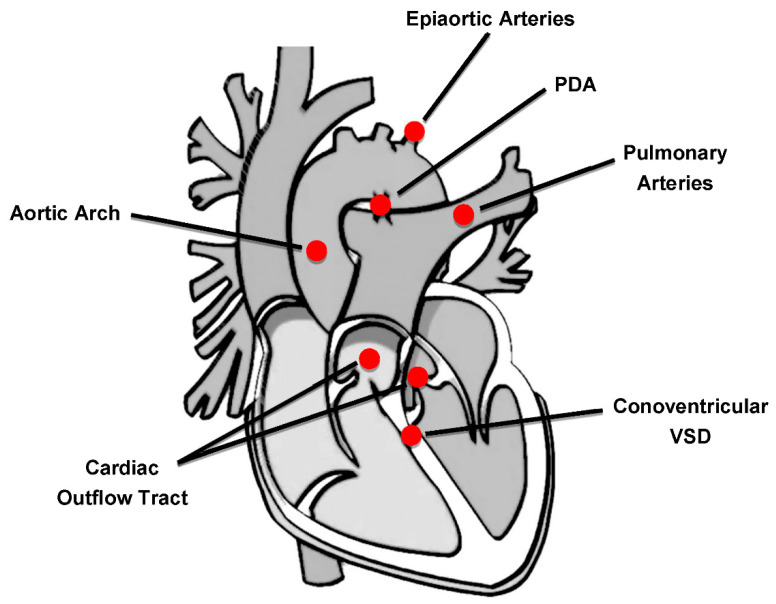
Cardiovascular regions involved in congenital heart defects of 22q11.2DS patients. PDA: patent ductus arteriosus; VSD: ventricular septal defect.

The 22q11.2 deletion syndrome (22q11.2DS) (OMIM 188400/192430), also known as DiGeorge syndrome or velocardiofacial syndrome, is the most frequent chromosomal microdeletion disorder in humans, with an estimated prevalence ranging from 1:3000 to 1:6000 live births [6,7]. The clinical phenotype is highly variable with multiorgan involvement, including CHDs, facial dysmorphisms, velopharyngeal anomalies, hypoparathyroidism, immunological disorders, developmental delay, and psychiatric manifestations [7]. CHDs represent one of the major features of this syndrome and the most common cardiac anomalies observed are conotruncal heart defects (CTDs), affecting the outflow tract and the aortic arch [8,9,10] (Figure 1).

Over time, the in-depth knowledge of the cardiac anatomy’s features, more commonly found in this genetic syndrome, led to improved cardiac surgical outcomes with a consequent increased survival during pediatric age and achievement of adulthood [11]. Nevertheless, CHDs, and in particular some cardiac malformations among others, are still the main cause of mortality (about 87%) in children with 22q11.2DS [12].

Moreover, the little data available on the adult population with 22q11.2DS report that the leading cause of mortality in this genetic syndrome are cardiovascular ones, such as sudden death and heart failure, not only in patients with major CHD but also in those without it [13,14].

As in other genetic syndromes, such as Down syndrome [15], Ellis-van Creveld syndrome [16], and Noonan syndrome [17], specific cardiac phenotypes have been delineated in patients with 22q11.2DS [8,18,19].

Several previous studies analyzed data on cardiac surgical outcome of patients with CTDs, including those with 22q11.2DS, showing that, over the years, with advances in medical and surgical treatment, the mortality rate after cardiac surgery has been reduced and the postsurgical outcome has been significantly improved [20,21,22,23]. Despite some previous reports which did not show any greater immediate surgical mortality in CTDs when associated to the deletion, the presence of a specific cardiac anatomy and the extracardiac comorbidities related to the genetic defect (e.g., immunodeficiency, hypocalcemia, pulmonary vascular reactivity, increased risk of bleeding, ENT malformations) could increase the risk of postoperative complications in 22q11.2DS population [24,25,26,27,28].

Indeed, in some subgroups of patients, the presence of 22q11.2DS appears to be related to a longer duration of postsurgical intensive care, increased mechanical ventilation and prolonged hemodynamic support [29,30,31,32]. Other determinants that seem to negatively affect the cardiac surgical outcome of patients with 22q11.2DS are prematurity and low body weight, frequently observed in this population [33,34].

It remains unclear whether cardiac surgery for CHDs in patients with 22q11.2DS is responsible for a worse neurological outcome. Some studies have demonstrated that patients with 22q11.2DS and CHDs have an unfavorable neurodevelopmental outcome than the non-syndromic counterparts, which appears to be more related to the presence of the chromosomal abnormality rather than to the underlying cardiac defect or surgical treatment [35,36]. Maharasingam and colleagues pointed out that a reduced developmental outcome in patients with 22q11.2DS was not only due to the repair of the concomitant CHD, but could be related to the genetic condition itself or to the interaction between the genetic syndrome and the possible perioperative effects of the cardiac surgery [37].

This review is an update of the literature of the last decades on the impact of 22q11.2DS on cardiac treatment for each of the most frequently associated CHDs, highlighting how the extensive knowledge of specific cardiac phenotypes and related extracardiac anomalies are critical, in order to provide the most suitable surgical approach and the most appropriate perioperative management to improve outcome and prognosis of these patients.

## 2. Congenital Heart Defects in 22q11.2 Deletion Syndrome

CHDs represent one of the most recurring features of 22q11.2DS, with a prevalence of 75–80% [6,8,9,38]. It is well established that the most common intracardiac CHDs associated with 22q11.2DS are CTDs, such as tetralogy of Fallot (ToF), pulmonary atresia with ventricular septal defect (PA-VSD), interrupted aortic arch (IAA), mainly type B, truncus arteriosus (TA), and conoventricular ventricular septal defect (VSD) [8,9,10,19,39]. In addition, anomalies of the aortic arch (AAA) and its branching are more frequently seen in this population, not only as isolated features but also in association with other CHDs, such as cervical aortic arch (CAA), double aortic arch (DAA), right-sided aortic arch (RAA) with aberrant subclavian arteries, and RAA with a vascular ring made by a left-sided ductus arising from the descending aorta [10,40].

Several prevalence studies of CTDs in persons with 22q11.2DS reported that ToF is the most frequent cardiac defect, occurring in about 20–45% of patients, followed by PA-VSD that is present in about 10–25%. Approximately 5–20% of patients with 22q11.2 have IAA, 5–10% TA and 10–50% VSD. In addition, AAA may also be associated with this syndrome with a prevalence of 10% [6,8,9,38] (Table 1).

Over the years, studies on cardiac phenotype allowed the identification of specific forms and subtypes of heart defects associated with this genetic syndrome [8,18,19]. Indeed, patients with 22q11.2DS more frequently show CTDs associated with additional cardiovascular anomalies, in a typical distinguishable pattern, which may complicate the cardiac anatomy, affecting surgical treatment and perioperative outcome [8,25,41,42,43].

Specifically, in individuals with 22q11.2DS, ToF may occur in association with hypoplasia or absent infundibular septum, absent pulmonary valve, discontinuity, diffuse hypoplasia or crossing of the pulmonary arteries (PAs) and AAA (such as RAA and CAA) with or without aberrant left subclavian artery [8,44,45,46]. Major aortopulmonary collateral arteries (MAPCAs) with hypoplasia and discontinuity of the PAs are common in PA-VSD [18,47].

A possible feature of TA in patients with 22q11.2DS is the discontinuity of PAs and AAAs (as IAA type B with RAA or DAA), and severe dysplasia with truncal valve stenosis [39,48]. Moreover, the presence of subarterial VSD associated with infundibular septal hypoplasia and aberrant right subclavian artery is suggestive of 22q11.2DS [49,50].

The aortic root dilation (ARD) is another cardiac finding reported among children and young adults with 22q11.2DS, isolated or associated with minor cardiovascular anomalies [51,52]. Nowadays, it is known that a progressive aortic root dilatation may be present in patients with CTDs, such as ToF, especially when associated with anomalies of the pulmonary valve and RAA, cardiovascular characteristics often found in 22q11.2DS population [53,54].

Currently, the clinical significance and the long-term implications of the isolated ARD in patients with 22q11.2DS are still unknown. Considering that, to date, there is no evidence of aortic dissection in the 22q11.2DS population and there are no surgical data on this aspect, it remains unclear whether the progression of dilatation may become significant over time.

The genotype–phenotype correlation between the microdeletion and the presence of CHDs is now well known, but the pathogenetic mechanisms leading to this strong association are still not fully understood. An increased risk of having a CHD in 22q11.2DS has been attributed primarily to the presence of the hemizygous deletion, and several authors have focused on the role of protein encoding genes within the critical deletion region. Multiple mouse model approaches have demonstrated that, within this region, TBX1 is the major transcription factor, mainly responsible for the clinical phenotype, including the cardiac ones [55,56,57,58,59]. Nonetheless, the high phenotypic variability, characteristics of this syndrome, seem to be related not only to the hemizygous deletion 22q11.2 or the size of the deleted region but also to the contribution of additional genetic factors outside the 22q11.2 region and/or environmental aspects acting as modifiers [60].

Zhao and colleagues have in fact recently provided compelling findings on genetic modifiers of CHDs in 22q11.2DS [61]. The authors found that the varying penetrance of CTDs in the 22q11.2DS population can be explained partly by common single nucleotide variants, located within the LCR22C-LCR22D intervals on the intact allele, which affect CRKL gene expression.

A miRNA-related mechanism was also observed in 22q11.2DS. Indeed, another gene that appears to be implicated in the cardiac phenotype of this syndrome is DGCR8, which encodes an RNA binding protein that is involved in miRNA biogenesis. In mouse models, inactivation of both DGCR8 alleles in neural crest cells has been proven to cause heart defects [62].

Understanding in more detail the pathogenic process leading to the presence of CHDs in those patients, may also allow to better comprehending the cause of coexisting comorbidities that could influence clinical and surgical outcome.

It is known that a significant percentage of patients with 22q11.2DS and CHD may require repeated cardiac operations, cardiac catheterization, and interventional procedures [11,25]. To date, however, it is not known whether adults with 22q11.2DS and CHDs are at greater risk of long-term complications or have higher need of reintervention than non-syndromic patients. At present, the timeline of the cardiac surgery (palliation, complete repair, reoperation) for persons with 22q11.2DS is the same as for non-syndromic patients with the same CHD, and there are no specific indications to a different timing of surgical approaches in this syndrome. Rather, the type of surgical technique can be modified and personally individualized, depending on the peculiar cardiac anatomy [21,22,23,24,25]. Therefore, 22q11.2DS patients with CHDs, like non-syndromic CHD population, require appropriate management and regular follow-up, specific for each cardiac lesion, throughout life, even after corrective surgery, to monitor any potential residual valve lesions, onset of outflow tract obstruction and/or endocarditis, evaluation of ventricular function, outbreak of arrhythmias, and the possible progression or appearance of aortic root dilation. Furthermore, given the increased risk of earlier mortality from cardiovascular causes, it seems appropriate that patients without congenital heart disease should also be followed-up periodically, especially if other comorbidities coexist [14,63,64,65].

## 3. Outcome of Cardiac Surgery in 22q11.2 Deletion Syndrome

### 3.1. Tetralogy of Fallot (ToF)

About 20–40% of children with 22q11.2DS suffer from ToF [6,8,9]. Of all patients with ToF, 10–15% have 22q11.2DS [41,66,67] (Table 1).

In about 50% of patients with 22q11.2DS, ToF recurs in association with particular cardiac anomalies: absent or hypoplastic infundibular septum, absent pulmonary valve, hypoplastic pulmonary arteries, and AAA (e.g., RAA or CAA) with or without aberrant subclavian arteries [8,44,45,46].

The appropriate choice of surgical techniques tailored on syndromic patients led to the accomplishment that 22q11.2DS is not a surgical risk factor in children with ToF [24]. Michielon and colleagues [21] demonstrated that 22q11.2DS was not a risk factor for primary surgical repair of ToF but the authors suggest that, in patients with ToF and normal pulmonary artery anatomy, a primary repair should be preferred instead of palliative procedures. The study showed that primary repair beyond neonatal age is the best surgical intervention in terms of lower mortality rates and higher time of freedom from reintervention. However, their study excluded patients with complex cardiac phenotype such as PA-VSD+/−MAPCAs, absent pulmonary valve, mitral-aortic discontinuity or associated atrioventricular septal defects.

The following study by Michielon et al. [22] confirmed that 22q11.2DS and other genetic syndromes (i.e., trisomy 21) are not independent risk factors for mortality after surgical repair of CTDs, including ToF. Indeed, long-term survival in 22q11.2DS was comparable with non-syndromic CTDs.

McDonald and colleagues [28] demonstrated that children with 22q11.2DS show higher postoperative complications after cardiac surgery. Length of stay (LOS) in intensive care setting and hospital stay were longer for the 22q11DS patients with correction for ToF, even if there is no significant difference among syndromic and non-syndromic patients, regarding length of mechanical ventilation and overall mortality. Moreover, the authors found a higher incidence of postsurgical complications, as fungal infection or wound infection, among children with genetic abnormalities.

However, early operative outcomes in a particular ToF subset can be affected by genotype, as demonstrated by Mercer Rosa et al. [30]. In fact, the subgroup of 22q11.2DS patients with ToF with pulmonary stenosis or PA who underwent the VSD closure, either as a single stage operation or preceded by an aortopulmonary shunt, showed longer cardiopulmonary bypass time and longer duration of intensive care.

Impact of genetics on perioperative outcomes has also been investigated by a recent study of the same group [68] who demonstrated, in a small cohort of survivors with ToF/MAPCAs, that 22q11.2DS had no significant impact on perioperative outcomes. The postoperative intensive care course was similar between syndromic and non-syndromic patients with no relevant difference in terms of several variables such as duration of mechanical ventilation, number of vasoactive medications or number of days of intensive care.

Moreover, interestingly, the authors did not find any significant difference in feeding status upon discharge, even though it is well known that patients with 22q11.2DS may have more feeding difficulties and can be discharged more commonly on gastric feeding.

### 3.2. Pulmonary Atresia with Ventricular Septal Defect +/− Major Aortopulmonary Collateral Arteries (PA-VSD+/−MAPCAs)

PA-VSD+/−MAPCAs is found in 10–25% of individuals with 22q11.2DS (Table 1), represents an extreme form of ToF and is characterized by a complete obstruction of the right ventricular outflow tract, and a variable number of systemic arteries that supply the pulmonary blood flow [8]. Approximately 40% of patients with ToF/PA/MAPCAs present a deletion of the chromosome 22q11.2 [69,70] (Table 1), and they show a typical and more complex pulmonary vascular phenotype [44], consisting of a higher occurrence of major aortopulmonary collaterals and hypoplastic pulmonary arteries.

The impact of genetics on perioperative and postoperative courses of patients with PA-VSD/MAPCAs has been the topic of numerous investigations.

In contrast to ToF in the context of 22q11.2DS, several studies demonstrated an increased mortality risk for patients with PA-VSD/MAPCAs and 22q11.2DS, underlying that the risk of mortality for PA-VSD was higher, particularly if associated with MAPCAs, probably due to a less favorable pulmonary vascular anatomy [24,33,69].

Carotti et al. [33] found a significant association of 22q11.2DS with mortality among patients treated for PA-VSD and demonstrated that the syndrome itself represents a strong independent variable affecting survival. Moreover, the authors speculated that the higher postoperative morbidity in 22q11.2DS patients could also be related to the defective immunologic status and to the increased infection susceptibility than to the cardiac anatomy. However, in the recent surgical era, survival seems to have improved.

Mahle et al. [70] showed that, in patients with PA-VSD, 22q11.2DS carries a significant risk for death related to surgical procedure, demonstrating that the five-year survival was 36% for patients with this syndrome versus 90% for non-syndromic patients. The authors did not find any significant discrepancy between syndromic and non-syndromic patients concerning the perioperative incidence of viral, bacterial, or fungal infections, and they suggested that the increased mortality among syndromic children was due to a less favorable pulmonary anatomic pattern.

Genotype affecting adverse perioperative outcome was also demonstrated by Kyburz and colleagues [26], who performed a prospective five-year multicenter study to outline the postsurgical death incidence of children with CTDs, in relation to the concomitant presence of genetic syndromes. The authors identified 22q11DS as a risk factor for immediate surgical mortality in patients with PA-VSD with a relative risk of 2.4 compared to non-syndromic patients as well as in patients with interrupted aortic arch.

A higher rate of postsurgical complications in 22q11.2DS patients was also reported by Ziolkowska and colleagues [27]. The authors revealed that the postoperative period for ToF and PA-VSD was complicated in the totality of patients with 22q11.2DS compared to a small proportion of patients without deletion.

A recent study by Koth and colleagues [29] confirmed previous observations. The authors investigated the role of 22q11.2DS in a cohort of patients with ToF/PA/MAPCAs who were treated surgically, with primary or revision of the unifocalization, with or without VSD closure, and they found that 22q11.2DS predisposes to a more complicated postoperative course. Syndromic patients more frequently needed delayed sternal closure, prolonged mechanical ventilation time, and extended intensive care and LOS due to the extensive nature of this surgery.

### 3.3. Truncus Arteriosus (TA)

TA can be associated with 22q11.2DS in up to 35% of cases [37,71,72]. Among patients with 22q11.2DS, the incidence of TA can rise up to 10% [8,38] (Table 1).

The reported data needed to be analyzed mindfully, as most of the studies were carried out over a prolonged period and therefore, some techniques could have updated, impacting their results over the study.

In terms of surgical timing, the current literature reports a younger age at correction in children with 22q11.2DS with TA, especially in the TA type 1, with the majority of interventions performed during the neonatal period [73].

In addition, some studies have pointed out the association between genetic syndromes and the need of unforeseen cardiac reoperations, without a definite explanation yet [74]. More specifically, regarding TA in 22q11DS, O’Byrne et al. showed a higher rate of reoperation, without a clear underlying etiopathology [31]. The postoperative period is largely more complicated when compared with non-syndromic patients, with the occurrence of pleural effusion, arrhythmias, and need for dialysis. Specifically, in terms of non-cardiac complications, Gupta et al. documented a similar rate of morbidity post TA correction with or without 22q11.2DS. Nonetheless, fungal and wound infections without fungal septicemia or positive blood cultures were more frequently documented in 22q11.2DS [33,75].

When compared to the control groups, some authors also reported a more common need of gastrostomy postoperatively, which could be related to the known feeding issues characterizing the syndrome itself. The time spent in the hospital prior to surgery was not influenced by the presence of the syndrome, but postoperatively, it was longer in 22q11DS subjects in most studies [31].

Consistently with a required longer total LOS, the 22q11DS population showed a greater use of resources, as a need for longer mechanical ventilation (twice as long as the control group), prolonged time of intensive care, and a more frequent use of consultation and medical treatment at discharge.

The incidence of non-cardiac complications was also greater. Hamzah and colleagues reported a postsurgical mortality rate from 6.9 to 11% [76,77,78]. Particularly, the authors highlighted significant in-hospital mortality (more than 10% of cases). The reported mortality rate showed a steady trend and, interestingly, no significant decrease was seen over the years of the study. Worse prognosis concerned neonates, especially in cases of prematurity (i.e., GA < 37 weeks) and/or low birth weight (<2500 g). These subgroups presented an increased odd risk mortality: OR = 1.95; 95% CI: 1.40–2.72; *p* < 0.001 and OR = 1.39; 95% CI: 0.95–2.03; *p* = 0.087, respectively. In neonates with 22q11.2DS undergoing TA repair, the operative course was longer and more complex, in relation to non-cardiac features related to the syndrome.

### 3.4. Interrupted Aortic Arch (IAA)

IAA occurs in about 5–20% of children with 22q11.2DS [8,9] (Table 1). In particular, the most frequent form of IAA associated with 22q11.2DS is the IAA type B (IAA-B), in which the interruption of the aortic arch is located between the left carotid artery and the left subclavian artery [38]. Of all patients with IAA-B, 50–80% have 22q11.2DS, representing one of the cardiac phenotypes more powerfully related to this genetic syndrome [43,50,67] (Table 1).

The typical intracardiac phenotype of the IAA in 22q11.2DS includes a hypoplastic and posteriorly deviated infundibular septum, particularly visible in type B anatomy [43,50]. A bicuspid aortic valve and/or aortic annulus hypoplasia can be associated. An aberrant right subclavian artery may also be present, particularly in association with IAA-B. In some cases, IAA presents with other complex cardiovascular abnormalities, such as obstructive lesions of the left ventricle outflow tract, particularly subaortic stenosis, which can complicate surgical management. Finally, although more rarely, an association with RAA or TA has also been described [43,50].

Some studies have previously shown that, although 22q11.2DS was not clearly acknowledged as an additional risk factor per se, many of its anatomical features could impact the surgical technique, contributing to increased mortality [24,79]. Anaclerio and colleagues pointed out that after treatment for IAA, immediate surgical mortality was higher in syndromic patients than in non-syndromic individuals with the same CHD, as well as for AP-VSD [24]. A following study on a large cohort of neonates with IAA demonstrated that in 22q11.2DS, specific anatomic features, such as a concomitant subaortic obstruction, was an incremental risk factor for mortality and affected initial left ventricular outflow tract procedures. Other interesting data revealed that the characteristics of the arch repair affected arch reintervention. Furthermore, the risk of mortality was increased if IAA was associated with TA [79].

In addition, a report by Kyburz and colleagues found a high risk of mortality and morbidity, mainly as the consequence of the severity of the CHD, but also influenced by the concomitant extracardiac anomalies, indicating a mortality rate of 30% for IAA [26].

These results have not been confirmed by a more recent retrospective study on a larger cohort of children undergoing neonatal repair for TA or IAA, comparing perioperative outcomes between individuals with 22q11DS and those without deletion. Indeed, the authors demonstrated that there were no significant differences in terms of immediate postsurgical mortality between syndromic and non-syndromic patients [31]. The longer and more complicated postoperative course in patients with 22q11.2DS appeared to be mainly related to the need for reoperations due to an increase in residual lesions and/or cardiovascular dysfunction, rather than to infections and immunodeficiency which were considered in other studies as risk factors for postoperative morbidity and mortality [26,28,30,70].

In particular, the association between the presence of 22q11.2DS and prolonged total LOS, longer time of mechanical ventilation and intensive care, and higher frequency of cardiac events was statistically significant in the subgroup of patients with IAA. Furthermore, the duration of cardiopulmonary bypass and deep hypothermic circulatory arrest was longer in patients with IAA than those with TA. Finally, the authors demonstrated that, compared to non-syndromic individuals with IAA, patients with 22q11.2DS and IAA required a greater number of subspecialty consultations and more medical treatments at discharge, while feeding problems did not affect the length of hospitalization [31].

McDonald and colleagues also reported that postsurgical intensive care unit hospitalization was longer in patients with 22q11.2DS undergoing surgery for IAA than in a non-syndromic group [28].

A very recent article demonstrated that the co-occurrence of simple and complex CHDs in patients with 22q11.2DS and IAA resulted in a higher risk of hospital complications (such as increased risk of gastrostomy tube placement and sepsis) compared with the non-syndromic counterpart [80].

## 4. Perioperative Management

Various authors highlighted a significant risk of mortality and morbidity in patients with 22q11.2DS when analyzing the postsurgical outcome and the possibility of a tailored pre-surgical management. This risk has been subsequently stratified for different eras of cardiac surgery, confirming a significant decrease during the recent years when compared to what was previously reported [32].

Nonetheless, patients with 22q11.2DS undergo surgical palliation more frequently than the complete surgical treatment (15 out of 40 cases reported by Kyburz et al., 2008) [26]. Severe postsurgical complications have been reported (more than 74% of cases) with a higher risk of reintervention (46%), secondary to significant residual cardiac findings (71%).

An additional extracardiac surgery (more than 40% of cases reported) was often a complication of the clinical outcome [26].

Different causes of death have been described in these patients. Even though mortality for cardiac illness remains most frequently reported (more than 54% of cases), immunological disorders sometimes worsen the outcome, particularly in patients younger than 2 years who still experience a worse postoperative outcome [26].

The patients with 22q11DS frequently need a prolonged time of mechanical ventilation postoperatively, with a consequent increased risk of lung infections. The same high risk is corroborated by a significant percentage of reintubation over the following day post operation. A dramatic risk of bacterial [32] or fungal infections, particularly evident as wound infections, has been reported [33].

Moreover, the role of hypocalcemia has been suggested, by some authors, for its impact on postsurgical outcomes. 22q11.2DS patients are well known to be prone to severe hypocalcemia, particularly in the neonatal period. In persons with 22q11.2DS, the high susceptibility to hypocalcemia, whether related to a preexisting condition of hypoparathyroidism and/or surgery-induced stress, is responsible for an increased risk of postoperative complications and mortality [81]. Yeoh and colleagues suggested that a major role of low calcium levels in the postsurgical hemodynamic instability or in the development of postoperative seizures might worsen the clinical outcome [32] (Table 2).

Ziolkowska and colleagues compared patients with CTDs, with and without 22q11.2DS, and underlined, in the syndrome, a greater risk for heart failure (*p* = 0.020), pneumonia (*p* < 0.001), sepsis (*p* = 0.001), respiratory failure (*p* = 0.028), prolonged intubation/tracheostomy (*p* = 0.014), laryngeal stridor (*p* < 0.001), and hypocalcemia (*p* = 0.015) [27].

Despite that the postsurgical outcome in these patients has vastly improved in the recent period [21,22], all the above-mentioned data still justify a complex postoperative period with complications to be expected [3].

Atallah et al. followed-up patients at 18–24 months and reported a lower neurodevelopmental outcome, and a higher motor and mental delay with lower IQ scores [36]. Therefore, over the most recent times, single centers have considered different strategies in order to reduce these increased risks. A longer duration of inotropic support or postoperatively antibiotic prophylaxis have been outlined by some authors when compared to non-syndromic subgroups.

Conversely, some other authors suggested the importance of a preoperative immunological evaluation of the T cell function. This information could indeed lead consultants and surgeons to a more specific postoperative antimicrobic management, in order to reduce complications [32] (Table 2).

Due to the tendency of 22q11.2DS patients to manifest thrombocytopenia and prolonged time of bleeding, a complete blood and platelet count should be perioperatively performed [32,82] (Table 2).

The use of previously filtered or irradiated red cells should be carefully considered to reduce graft versus hosting disease in the time of transfusion or for aortic cross-clamping [32].

Furthermore, a careful imaging study of some anatomical features, often found in 22q11.2DS (such as pharyngeal malformations, cervical spine anomalies, neck vessels abnormalities, the possible presence of a vascular ring), can allow to reducing the risk of complications during surgery [10,60,83,84] (Table 2).

Finally, targeted therapies (e.g., vasopressors and/or bronchodilators) may be required due to an increased risk of hypotension, related to vasomotor instability, and airway over-reactivity [85,86] (Table 2).

## 5. Conclusions

Over the years, the deep knowledge of the anatomic cardiac phenotype allowed the adaptation of routine surgical techniques and has greatly improved surgical results in patients with 22q11.2DS.

To date, although 22q11.2DS does not represent an independent risk factor for mortality after cardiac surgery, the short-term surgical outcome may vary for subgroups of patients with specific cardiac phenotypes, especially in patients with PA-VSD and MAPCAs due to unfavorable pulmonary vascular anatomy. Different surgical outcomes have been reported by different centers in the last decades. Certainly, the reason is multifactorial depending on variables such as the sample size of population of patients, age and weight of patients at the moment of the operation, primary or staged surgical approach, metabolic abnormalities, and undetected infections. Considering that the presence of this genetic syndrome and related comorbidities may adversely affect the postoperative course, results of cardiac surgery of the more recent studies are extremely relevant to preoperative counseling, to guide surgical decisions, and for tailored perioperative management.

With a deeper understanding of cardiac phenotypes, perioperative management and genotype–phenotype correlations of patients with 22q11.2DS, it will be possible to offer to families, even in the prenatal period, appropriate counseling in terms of prognosis, to plan surgical procedures, and to define postoperative management strategies in order to improve outcomes and resource management when taking care of this highly complex population of patients.

## Figures and Tables

**Table 1:** Prevalence of the most frequent congenital heart diseases observed in 22q11.2DS.

Congenital Heart Diseases	% 22q11.2DS Patients ^(^*^)^	% Cases Associated with 22q11.2DS ^(^*^)^
Tetralogy of Fallot	20–45%	10–15%
Pulmonary atresia + VSD	10–25%	30–45%
Aortic arch interruption	5–20%	50–80%
Truncus arteriosus	5–10%	30–50%
Conoventricular VSD	10–50%	5%
Isolated aortic arch anomalies	10%	25%

^(^*^)^ The reported prevalence is based on a literature review of large cohorts of patients with 22q11.2DS (Botto et al., 2003; Marino et al., 2001; Momma et al., 2010; Matsuoka et al., 1998; Digilio et al., 1996; Goldmuntz et al., 1998; Peyvandi et al., 2013).

**Table 2:** Perioperative management of 22q11.2DS patients.

Problem	Perioperative Management ^(^*^)^
Immunological disorders	- Pre-operative check of immunological status - Antibiotic and antifungal prophylaxis - Transfusion of filtered/irradiated and cytomegalovirus-seronegative blood products
Hypocalcemia	- Peri-operative check of calcium levels - Pharmacological prophylaxis for patients affected by hypocalcemia-induced seizures
Thrombocytopenia	- Peri-operative check of platelet count
Velopharyngeal, upper cervical spine, and neck vessels abnormalities	- Pre-operative multispecialist assessment (anesthesiologist, otolaryngologist, and plastic surgeon) - MRI (if needed, in the suspicion of vascular rings or to evaluate cervical spine anomalies and vascular neck malformations)
Risk of pulmonary hyper-reactivity and vasomotor instability	- Peri-operative administration of targeted therapies, if needed (bronchodilators and/or vasopressors)

^(^*^)^ (Fung et al., 2015; Yeoh et al., 2014; Shen et al., 2011; Kato et al., 2003; Stransky et al., 2015; Sacca et al., 2017, McElhinney et al., 2001; Shashi et al., 2003; Ackerman et al., 2001).

## Data Availability

Not applicable.

## References

[1] Donofrio M.T., Moon-Grady A.J., Hornberger L.K., Copel J.A., Sklansky M.S., Abuhamad A., Cuneo B.F., Huhta J.C., Jonas R.A., Krishnan A. (2014). Diagnosis and treatment of fetal cardiac disease: A scientific statement from the American Heart Association. Circulation.

[2] Jacobs J.P., He X., Mayer J.E., Austin E.H., Quintessenza J.A., Karl T.R., Vricella L., Mavroudis C., O’Brien S.M., Pasquali S.K. (2016). Mortality Trends in Pediatric and Congenital Heart Surgery: An Analysis of The Society of Thoracic Surgeons Congenital Heart Surgery Database. Ann. Thorac. Surg..

[3] Calcagni G., Pugnaloni F., Digilio M.C., Unolt M., Putotto C., Niceta M., Baban A., Piceci Sparascio F., Drago F., De Luca A. (2021). Cardiac Defects and Genetic Syndromes: Old Uncertainties and New Insights. Genes.

[4] Marino B., Digilio M.C. (2000). Congenital heart disease and genetic syndromes: Specific correlation between cardiac phenotype and genotype. Cardiovasc. Pathol..

[5] Formigari R., Michielon G., Digilio M.C., Piacentini G., Carotti A., Giardini A., Di Donato R.M., Marino B. (2009). Genetic syndromes and congenital heart defects: How is surgical management affected?. Eur. J. Cardiothorac. Surg..

[6] Botto L.D., May K., Fernhoff P.M., Correa A., Coleman K., Rasmussen S.A., Merritt R.K., O’Leary L.A., Wong L.Y., Elixson E.M. (2003). A population-based study of the 22q11.2 deletion: Phenotype, incidence, and contribution to major birth defects in the population. Pediatrics.

[7] McDonald-McGinn D.M., Sullivan K.E., Marino B., Philip N., Swillen A., Vorstman J.A., Zackai E.H., Emanuel B.S., Vermeesch J.R., Morrow B.E. (2015). 22q11.2 Deletion Syndrome. Nat. Rev. Dis. Primers.

[8] Marino B., Digilio M.C., Toscano A., Anaclerio S., Giannotti A., Feltri C., de Ioris M.A., Angioni A., Dallapiccola B. (2001). Anatomic patterns of conotruncal defects associated with deletion 22q11. Genet. Med..

[9] Momma K. (2010). Cardiovascular anomalies associated with chromosome 22q11.2 deletion syndrome. Am. J. Cardiol..

[10] McElhinney D.B., Clark B.J., Weinberg P.M., Kenton M.L., McDonald-McGinn D., Driscoll D.A., Zackai E.H., Goldmuntz E. (2001). Association of chromosome 22q11 deletion with isolated anomalies of aortic arch laterality and branching. J. Am. Coll. Cardiol..

[11] Lin A.E., Basson C.T., Goldmuntz E., Magoulas P.L., McDermott D.A., McDonald-McGinn D.M., McPherson E., Morris C.A., Noonan J., Nowak C. (2008). Adults with genetic syndromes and cardiovascular abnormalities: Clinical history and management. Genet. Med..

[12] Repetto G.M., Guzman M.L., Delgado I., Loyola H., Palomares M., Lay-Son G., Vial C., Benavides F., Espinoza K., Alvarez P. (2014). Case fatality rate and associated factors in patients with 22q11 microdeletion syndrome: A retrospective cohort study. BMJ Open.

[13] Bassett A.S., Chow E.W., Husted J., Hodgkinson K.A., Oechslin E., Harris L., Silversides C. (2009). Premature death in adults with 22q11.2 deletion syndrome. J. Med. Genet..

[14] Van L., Heung T., Graffi J., Ng E., Malecki S., Van Mil S., Boot E., Corral M., Chow E.W.C., Hodgkinson K.A. (2019). All-cause mortality and survival in adults with 22q11.2 deletion syndrome. Genet. Med..

[15] Marino B., Vairo U., Corno A., Nava S., Guccione P., Calabrò R., Marcelletti C. (1990). Atrioventricular canal in Down syndrome. Prevalence of associated cardiac malformations compared with patients without Down syndrome. Am. J. Dis. Child..

[16] Levin S.E., Dansky R., Milner S., Benatar A., Govendrageloo K., du Plessis J. (1995). Atrioventricular septal defect and type A postaxial polydactyly without other major associated anomalies: A specific association. Pediatr. Cardiol..

[17] Pierpont M.E., Digilio M.C. (2018). Cardiovascular disease in Noonan syndrome. Curr. Opin. Pediatr..

[18] Anaclerio S., Marino B., Carotti A., Digilio M.C., Toscano A., Gitto P., Giannotti A., Di Donato R., Dallapiccola B. (2001). Pulmonary atresia with ventricular septal defect: Prevalence of deletion 22q11 in the different anatomic patterns. Ital. Heart J..

[19] Toscano A., Anaclerio S., Digilio M.C., Giannotti A., Fariello G., Dallapiccola B., Marino B. (2002). Ventricular septal defect and deletion of chromosome 22q11: Anatomical types and aortic arch anomalies. Eur. J. Pediatr..

[20] Marmon L.M., Balsara R.K., Chen R., Dunn J.M. (1984). Congenital cardiac anomalies associated with the DiGeorge syndrome: A neonatal experience. Ann. Thorac. Surg..

[21] Michielon G., Marino B., Formigari R., Gargiulo G., Picchio F., Digilio M.C., Anaclerio S., Oricchio G., Sanders S.P., Di Donato R.M. (2006). Genetic syndromes and outcome after surgical correction of tetralogy of Fallot. Ann. Thorac. Surg..

[22] Michielon G., Marino B., Oricchio G., Digilio M.C., Iorio F., Filippelli S., Placidi S., Di Donato R.M. (2009). Impact of DEL22q11, trisomy 21, and other genetic syndromes on surgical outcome of conotruncal heart defects. J. Thorac. Cardiovasc. Surg..

[23] Simsic J.M., Coleman K., Maher K.O., Cuadrado A., Kirshbom P.M. (2009). Do neonates with genetic abnormalities have an increased morbidity and mortality following cardiac surgery?. Congenit. Heart Dis..

[24] Anaclerio S., Di Ciommo V., Michielon G., Digilio M.C., Formigari R., Picchio F.M., Gargiulo G., Di Donato R., De Ioris M.A., Marino B. (2004). Conotruncal heart defects: Impact of genetic syndromes on immediate operative mortality. Ital. Heart J..

[25] Carotti A., Digilio M.C., Piacentini G., Saffirio C., Di Donato R.M., Marino B. (2008). Cardiac defects and results of cardiac surgery in 22q11.2 deletion syndrome. Dev. Disabil. Res. Rev..

[26] Kyburz A., Bauersfeld U., Schinzel A., Riegel M., Hug M., Tomaske M., Valsangiacomo Buchel E.R. (2008). The fate of children with microdeletion 22q11.2 syndrome and congenital heart defect: Clinical course and cardiac outcome. Pediatr. Cardiol..

[27] Ziolkowska L., Kawalec W., Turska-Kmiec A., Krajewska-Walasek M., Brzezinska-Rajszys G., Daszkowska J., Maruszewski B., Burczynski P. (2008). Chromosome 22q11.2 microdeletion in children with conotruncal heart defects: Frequency, associated cardiovascular anomalies, and outcome following cardiac surgery. Eur. J. Pediatr..

[28] McDonald R., Dodgen A., Goyal S., Gossett J.M., Shinkawa T., Uppu S.C., Blanco C., Garcia X., Bhutta A.T., Imamura M. (2013). Impact of 22q11.2 deletion on the postoperative course of children after cardiac surgery. Pediatr. Cardiol..

[29] Koth A., Sidell D., Bauser-Heaton H., Wise-Faberowski L., Hanley F.L., McElhinney D.B., Asija R. (2019). Deletion of 22q11 chromosome is associated with postoperative morbidity after unifocalisation surgery. Cardiol. Young.

[30] Mercer-Rosa L., Pinto N., Yang W., Tanel R., Goldmuntz E. (2013). 22q11.2 Deletion syndrome is associated with perioperative outcome in tetralogy of Fallot. J. Thorac. Cardiovasc. Surg..

[31] O’Byrne M.L., Yang W., Mercer-Rosa L., Parnell A.S., Oster M.E., Levenbrown Y., Tanel R.E., Goldmuntz E. (2014). 22q11.2 Deletion syndrome is associated with increased perioperative events and more complicated postoperative course in infants undergoing infant operative correction of truncus arteriosus communis or interrupted aortic arch. J. Thorac. Cardiovasc. Surg..

[32] Yeoh T.Y., Scavonetto F., Hamlin R.J., Burkhart H.M., Sprung J., Weingarten T.N. (2014). Perioperative management of patients with DiGeorge syndrome undergoing cardiac surgery. J. Cardiothorac. Vasc. Anesth..

[33] Carotti A., Albanese S.B., Filippelli S., Rava L., Guccione P., Pongiglione G., Di Donato R.M. (2010). Determinants of outcome after surgical treatment of pulmonary atresia with ventricular septal defect and major aortopulmonary collateral arteries. J. Thorac. Cardiovasc. Surg..

[34] Besseau-Ayasse J., Violle-Poirsier C., Bazin A., Gruchy N., Moncla A., Girard F., Till M., Mugneret F., Coussement A., Pelluard F. (2014). A French collaborative survey of 272 fetuses with 22q11.2 deletion: Ultrasound findings, fetal autopsies and pregnancy outcomes. Prenat. Diagn..

[35] Swillen A., Feys H., Adriaens T., Nelissen L., Mertens L., Gewillig M., Devriendt K., Fryns J.P. (2005). Early motor development in young children with 22q.11 deletion syndrome and a conotruncal heart defect. Dev. Med. Child. Neurol..

[36] Atallah J., Joffe A.R., Robertson C.M., Leonard N., Blakley P.M., Nettel-Aguirre A., Sauve R.S., Ross D.B., Rebeyka I.M. (2007). Western Canadian Complex Pediatric Therapies Project Follow-up Group Two-year general and neurodevelopmental outcome after neonatal complex cardiac surgery in patients with deletion 22q11.2: A comparative study. J. Thorac. Cardiovasc. Surg..

[37] Maharasingam M., Ostman-Smith I., Pike M.G. (2003). A cohort study of neurodevelopmental outcome in children with DiGeorge syndrome following cardiac surgery. Arch. Dis. Child..

[38] Matsuoka R., Kimura M., Scambler P.J., Morrow B.E., Imamura S., Minoshima S., Shimizu N., Yamagishi H., Joh-o K., Watanabe S. (1998). Molecular and clinical study of 183 patients with conotruncal anomaly face syndrome. Hum. Genet..

[39] McElhinney D.B., Driscoll D.A., Levin E.R., Jawad A.F., Emanuel B.S., Goldmuntz E. (2003). Chromosome 22q11 deletion in patients with ventricular septal defect: Frequency and associated cardiovascular anomalies. Pediatrics.

[40] Momma K., Matsuoka R., Takao A. (1999). Aortic arch anomalies associated with chromosome 22q11 deletion (CATCH 22). Pediatr. Cardiol..

[41] Digilio M.C., Marino B., Giannotti A., Novelli G., Dallapiccola B. (1997). Conotruncal heart defects and chromosome 22q11 microdeletion. J. Pediatr..

[42] Goldmuntz E., Clark B.J., Mitchell L.E., Jawad A.F., Cuneo B.F., Reed L., McDonald-McGinn D., Chien P., Feuer J., Zackai E.H. (1998). Frequency of 22q11 deletions in patients with conotruncal defects. J. Am. Coll. Cardiol..

[43] Marino B., Digilio M.C., Persiani M., Di Donato R., Toscano A., Giannotti A., Dallapiccola B. (1999). Deletion 22q11 in patients with interrupted aortic arch. Am. J. Cardiol..

[44] Chessa M., Butera G., Bonhoeffer P., Iserin L., Kachaner J., Lyonnet S., Munnich A., Sidi D., Bonnet D. (1998). Relation of genotype 22q11 deletion to phenotype of pulmonary vessels in tetralogy of Fallot and pulmonary atresia-ventricular septal defect. Heart.

[45] Galindo A., Gutierrez-Larraya F., Martinez J.M., Del Rio M., Graneras A., Velasco J.M., Puerto B., Gratacos E. (2006). Prenatal diagnosis and outcome for fetuses with congenital absence of the pulmonary valve. Ultrasound Obs. Gynecol..

[46] Babaoglu K., Altun G., Binnetoglu K., Donmez M., Kayabey O., Anik Y. (2013). Crossed pulmonary arteries: A report on 20 cases with an emphasis on the clinical features and the genetic and cardiac abnormalities. Pediatr. Cardiol..

[47] Momma K., Kondo C., Matsuoka R. (1996). Tetralogy of Fallot with pulmonary atresia associated with chromosome 22q11 deletion. J. Am. Coll. Cardiol..

[48] Marino B., Digilio M.C., Dallapiccola B. (1998). Severe truncal valve dysplasia: Association with DiGeorge syndrome?. Ann. Thorac. Surg..

[49] Marino B., Digilio M.C., Toscano A., Dallapiccola B. (2000). Deficiency of the infundibular septum in patients with interrupted aortic arch and del 22q11. Cardiol. Young.

[50] Momma K., Ando M., Matsuoka R., Joo K. (1999). Interruption of the aortic arch associated with deletion of chromosome 22q11 is associated with a subarterial and doubly committed ventricular septal defect in Japanese patients. Cardiol. Young.

[51] John A.S., McDonald-McGinn D.M., Zackai E.H., Goldmuntz E. (2009). Aortic root dilation in patients with 22q11.2 deletion syndrome. Am. J. Med. Genet. A.

[52] De Rinaldis C.P., Butensky A., Patel S., Edman S., Wasserman M., McGinn D.E., Bailey A., Zackai E.H., Crowley T.B., McDonald-McGinn D.M. (2021). Aortic Root Dilation in Patients with 22q11.2 Deletion Syndrome without Intracardiac Anomalies. Pediatr. Cardiol..

[53] Niwa K. (2018). Aortic dilatation in complex congenital heart disease. Cardiovasc. Diagn. Ther..

[54] John A.S., Rychik J., Khan M., Yang W., Goldmuntz E. (2014). 22q11.2 deletion syndrome as a risk factor for aortic root dilation in tetralogy of Fallot. Cardiol. Young.

[55] Merscher S., Funke B., Epstein J.A., Heyer J., Puech A., Lu M.M., Xavier R.J., Demay M.B., Russell R.G., Factor S. (2001). TBX1 is responsible for cardiovascular defects in velo-cardio-facial/DiGeorge syndrome. Cell.

[56] Lindsay E.A., Vitelli F., Su H., Morishima M., Huynh T., Pramparo T., Jurecic V., Ogunrinu G., Sutherland H.F., Scambler P.J. (2001). Tbx1 haploinsufficieny in the DiGeorge syndrome region causes aortic arch defects in mice. Nature.

[57] Xu H., Morishima M., Wylie J.N., Schwartz R.J., Bruneau B.G., Lindsay E.A., Baldini A. (2004). Tbx1 has a dual role in the morphogenesis of the cardiac outflow tract. Development.

[58] Yagi H., Furutani Y., Hamada H., Sasaki T., Asakawa S., Minoshima S., Ichida F., Joo K., Kimura M., Imamura S. (2003). Role of TBX1 in human del22q11.2 syndrome. Lancet.

[59] Mastromoro G., Calcagni G., Versacci P., Putotto C., Chinali M., Lambiase C., Unolt M., Pelliccione E., Anaclerio S., Caprio C. (2019). Left pulmonary artery in 22q11.2 deletion syndrome. Echocardiographic evaluation in patients without cardiac defects and role of Tbx1 in mice. PLoS ONE.

[60] Goldmuntz E. (2020). 22q11.2 Deletion Syndrome and Congenital Heart Disease. Am. J. Med. Genet. C Semin. Med. Genet..

[61] Zhao Y., Diacou A., Johnston H.R., Musfee F.I., McDonald-McGinn D.M., McGinn D., Crowley T.B., Repetto G.M., Swillen A., Breckpot J. (2020). Complete Sequence of the 22q11.2 Allele in 1,053 Subjects with 22q11.2 Deletion Syndrome Reveals Modifiers of Conotruncal Heart Defects. Am. J. Hum. Genet..

[62] Chapnik E., Sasson V., Blelloch R., Hornstein E. (2012). Dgcr8 controls neural crest cells survival in cardiovascular development. Dev. Biol..

[63] Bassett A.S., McDonald-McGinn D.M., Devriendt K., Digilio M.C., Goldenberg P., Habel A., Marino B., Oskarsdottir S., Philip N., Sullivan K. (2011). International 22q11.2 Deletion Syndrome Consortium Practical guidelines for managing patients with 22q11.2 deletion syndrome. J. Pediatr..

[64] Fung W.L., Butcher N.J., Costain G., Andrade D.M., Boot E., Chow E.W., Chung B., Cytrynbaum C., Faghfoury H., Fishman L. (2015). Practical guidelines for managing adults with 22q11.2 deletion syndrome. Genet. Med..

[65] Unolt M., Versacci P., Anaclerio S., Lambiase C., Calcagni G., Trezzi M., Carotti A., Crowley T.B., Zackai E.H., Goldmuntz E. (2018). Congenital heart diseases and cardiovascular abnormalities in 22q11.2 deletion syndrome: From well-established knowledge to new frontiers. Am. J. Med. Genet. A.

[66] Digilio M.C., Marino B., Grazioli S., Agostino D., Giannotti A., Dallapiccola B. (1996). Comparison of occurrence of genetic syndromes in ventricular septal defect with pulmonic stenosis (classic tetralogy of Fallot) versus ventricular septal defect with pulmonic atresia. Am. J. Cardiol..

[67] Peyvandi S., Ingall E., Woyciechowski S., Garbarini J., Mitchell L.E., Goldmuntz E. (2014). Risk of congenital heart disease in relatives of probands with conotruncal cardiac defects: An evaluation of 1620 families. Am. J. Med. Genet. A.

[68] Mercer-Rosa L., Elci O.U., Pinto N.M., Tanel R.E., Goldmuntz E. (2018). 22q11.2 Deletion Status and Perioperative Outcomes for Tetralogy of Fallot with Pulmonary Atresia and Multiple Aortopulmonary Collateral Vessels. Pediatr. Cardiol..

[69] Carotti A., Di Donato R.M., Squitieri C., Guccione P., Catena G. (1998). Total repair of pulmonary atresia with ventricular septal defect and major aortopulmonary collaterals: An integrated approach. J. Thorac. Cardiovasc. Surg..

[70] Mahle W.T., Crisalli J., Coleman K., Campbell R.M., Tam V.K., Vincent R.N., Kanter K.R. (2003). Deletion of chromosome 22q11.2 and outcome in patients with pulmonary atresia and ventricular septal defect. Ann. Thorac. Surg..

[71] Momma K., Ando M., Matsuoka R. (1997). Truncus arteriosus communis associated with chromosome 22q11 deletion. J. Am. Coll. Cardiol..

[72] Marino B., Digilio M.C., Toscano A. (2002). Common arterial trunk, DiGeorge syndrome and microdeletion 22q11. Prog. Ped Card.

[73] Ghimire L.V., Devoe C., Moon-Grady A.J. (2020). 22q11.2 Deletion Status Influences Resource Utilization in Infants Requiring Repair of Tetralogy of Fallot and Common Arterial Trunk. Pediatr. Cardiol..

[74] Alsoufi B., Gillespie S., Mahle W.T., Deshpande S., Kogon B., Maher K., Kanter K. (2016). The Effect of Noncardiac and Genetic Abnormalities on Outcomes Following Neonatal Congenital Heart Surgery. Semin. Thorac. Cardiovasc. Surg..

[75] Gupta S.K., Aggarwal A., Shaw M., Gulati G.S., Kothari S.S., Ramakrishnan S., Saxena A., Devagourou V., Talwar S., Sharma S. (2020). Clarifying the anatomy of common arterial trunk: A clinical study of 70 patients. Eur. Heart J. Cardiovasc. Imaging.

[76] Hamzah M., Othman H.F., Daphtary K., Komarlu R., Aly H. (2020). Outcomes of truncus arteriosus repair and predictors of mortality. J. Card. Surg..

[77] Mastropietro C.W., Amula V., Sassalos P., Buckley J.R., Smerling A.J., Iliopoulos I., Riley C.M., Jennings A., Cashen K., Narasimhulu S.S. (2019). Collaborative Research in Pediatric Cardiac Intensive Care Investigators Characteristics and operative outcomes for children undergoing repair of truncus arteriosus: A contemporary multicenter analysis. J. Thorac. Cardiovasc. Surg..

[78] Russell H.M., Pasquali S.K., Jacobs J.P., Jacobs M.L., O’Brien S.M., Mavroudis C., Backer C.L. (2012). Outcomes of repair of common arterial trunk with truncal valve surgery: A review of the society of thoracic surgeons congenital heart surgery database. Ann. Thorac. Surg..

[79] McCrindle B.W., Tchervenkov C.I., Konstantinov I.E., Williams W.G., Neirotti R.A., Jacobs M.L., Blackstone E.H., Congenital Heart Surgeons Society (2005). Risk factors associated with mortality and interventions in 472 neonates with interrupted aortic arch: A Congenital Heart Surgeons Society study. J. Thorac. Cardiovasc. Surg..

[80] Sanchez Mejia A.A., Cambronero N., Dongarwar D., Salihu H.M., Vigil-Mallette M.A., Garcia B.Y., Morris S.A. (2022). Hospital Outcomes among Infants with Interrupted Aortic Arch with Simple and Complex Associated Heart Defects. Am. J. Cardiol..

[81] Shen L., Gu H., Wang D., Yang C., Xu Z., Jing H., Jiang Y., Ding Y., Hou H., Ge Z. (2011). Influence of chromosome 22q11.2 microdeletion on postoperative calcium level after cardiac-correction surgery. Pediatr. Cardiol..

[82] Kato T., Kosaka K., Kimura M., Imamura S., Yamada O., Iwai K., Matsuoka R. (2003). Thrombocytopenia in patients with 22q11.2 deletion syndrome and its association with glycoprotein Ib-beta. Genet. Med..

[83] Stransky C., Basta M., McDonald-McGinn D.M., Solot C.B., Drummond D., Zackai E., LaRossa D., Kirschner R., Jackson O. (2015). Perioperative risk factors in patients with 22q11.2 deletion syndrome requiring surgery for velopharyngel dysfunction. Cleft Palate Craniofac. J..

[84] Sacca R., Zur K.B., Crowley T.B., Zackai E.H., Valverde K.D., McDonald-McGinn D.M. (2017). Association of airway abnormalities with 22q11.2 deletion syndrome. Int. J. Pediatr. Otorhinolaryngol..

[85] Shashi V., Berry M.N., Hines M.H. (2003). Vasomotor instability in neonates with chromosome 22q11 deletion syndrome. Am. J. Med. Genet. A.

[86] Ackerman M.J., Wylam M.E., Feldt R.H., Porter C.J., Dewald G., Scanlon P.D., Driscoll D.J. (2001). Pulmonary atresia with ventricular septal defect and persistent airway hyperresponsiveness. J. Thorac. Cardiovasc. Surg..

